# Torque expression in conventional and self-ligating orthodontic brackets: An *in vitro* study

**DOI:** 10.1590/0103-644020266914

**Published:** 2026-08-24

**Authors:** Martha de Abreu Avila, José Guilherme Neves, Américo Bortolazzo Correr, Milton Santamaria-Junior, Eduardo Almada, Lourenço Correr-Sobrinho, Ana Rosa Costa

**Affiliations:** 1 Department of Orthodontics, Fundação Hermínio Ometto - FHO, Araras, SP, Brazil; 2 Department of Restorative Dentistry, Dental Materials Division, Piracicaba Dental School, UNICAMP, State University of Campinas, Piracicaba, SP, Brazil; 3 Department of Health, Orthodontics and Pediatric Dentistry, Piracicaba Dental School, UNICAMP, State University of Campinas, Piracicaba, SP, Brazil

**Keywords:** Orthodontic brackets, metal brackets, torque expression, SEM/EDS

## Abstract

This study assessed torque expression at different angulations of conventional, interactive, and passive self-ligating brackets, with or without metal ligatures, and evaluated surface morphology and chemical composition (%). All brackets (Roth prescription, 0.022" x 0.028" slot) were fixed to round acrylic cylinders with cyanoacrylate adhesive. Five hundred segments of rectangular archwires (0.019" x 0.025") were bent and welded (20 mm x 10 mm), then fixed to the brackets either with or without 0.010" metal ligatures in ten experimental groups (n = 10). The assembled specimens were then mounted on a universal testing machine, and a vertical force was applied to the free end of the archwire, resulting in a 10 mm displacement. Mean torque values (N·mm) at specified torque angles of 0° to 25°, as well as the mean torque angles (°) corresponding to 5 and 20 N·mm, were analyzed using ANOVA and Tukey’s test (α = 0.05). Bracket design and chemical composition were examined by SEM/EDS. Slot angulation and dimensional measurements were evaluated using ImageJ. Passive self-ligating brackets, without and with ligatures, showed the smallest torque angles, statistically smaller than those of the other groups (p < 0.001). Bracket clips were composed predominantly of nickel and titanium. Passive self-ligating brackets, either ligated or not, showed the highest torque expression at both 5 and 20 N·mm. Overall, torque expression was influenced more by the dimensional characteristics of brackets and rectangular archwires than by the type of ligature used. Clinically, passive self-ligating brackets may facilitate earlier torque expression during orthodontic treatment.

## Resumo

Este estudo avaliou a expressão de torque em diferentes angulações de braquetes metálicos convencionais, autoligados ativos e passivos, com ou sem ligaduras metálicas, bem como a morfologia superficial e a composição química (%) dos braquetes. Os braquetes (prescrição Roth e slot de 0,022" x 0,028") foram fixados com cianoacrilato em cilindros acrílicos. Quinhentos segmentos de fios ortodônticos retangulares (0,019" x 0,025") foram dobrados e soldados (20 mm x 10 mm) e fixados aos braquetes com ou sem ligaduras metálicas (0,010") nos dez grupos experimentais (n=10). Os segmentos foram fixados em uma máquina de ensaio universal, e uma força vertical foi aplicada à extremidade livre do fio ortodôntico, promovendo um deslocamento de 10 mm. Os valores médios de torque (N·mm ) em ângulos de torque específicos de 0° a 25°, bem como os ângulos médios de torque (°) correspondentes a 5 N·mm e 20 N·mm, foram submetidos à ANOVA seguida do teste de Tukey (α = 0,05). O desenho e a composição química dos braquetes foram avaliados por MEV/EDS. A angulação e as medidas do *slot* foram determinadas utilizando o *software* ImageJ. Os braquetes autoligáveis passivos, com e sem ligaduras, apresentaram os menores ângulos de torque, estatisticamente inferiores ao dos outros grupos (p < 0,001). De modo geral, os clips dos braquetes são compostos predominantemente de níquel e titânio (NiTi). Braquetes autoligáveis ​​passivos, com ou sem ligadura, apresentaram a melhor expressão de torque em 5 e 20 N·mm. As dimensões dos braquetes e dos arcos retangulares exercem maior influência na expressão do torque do que o tipo de ligadura utilizada. Clinicamente, esses achados sugerem que braquetes autoligáveis passivos podem favorecer uma expressão de torque mais precoce durante o tratamento ortodôntico**.**

**Figure:**
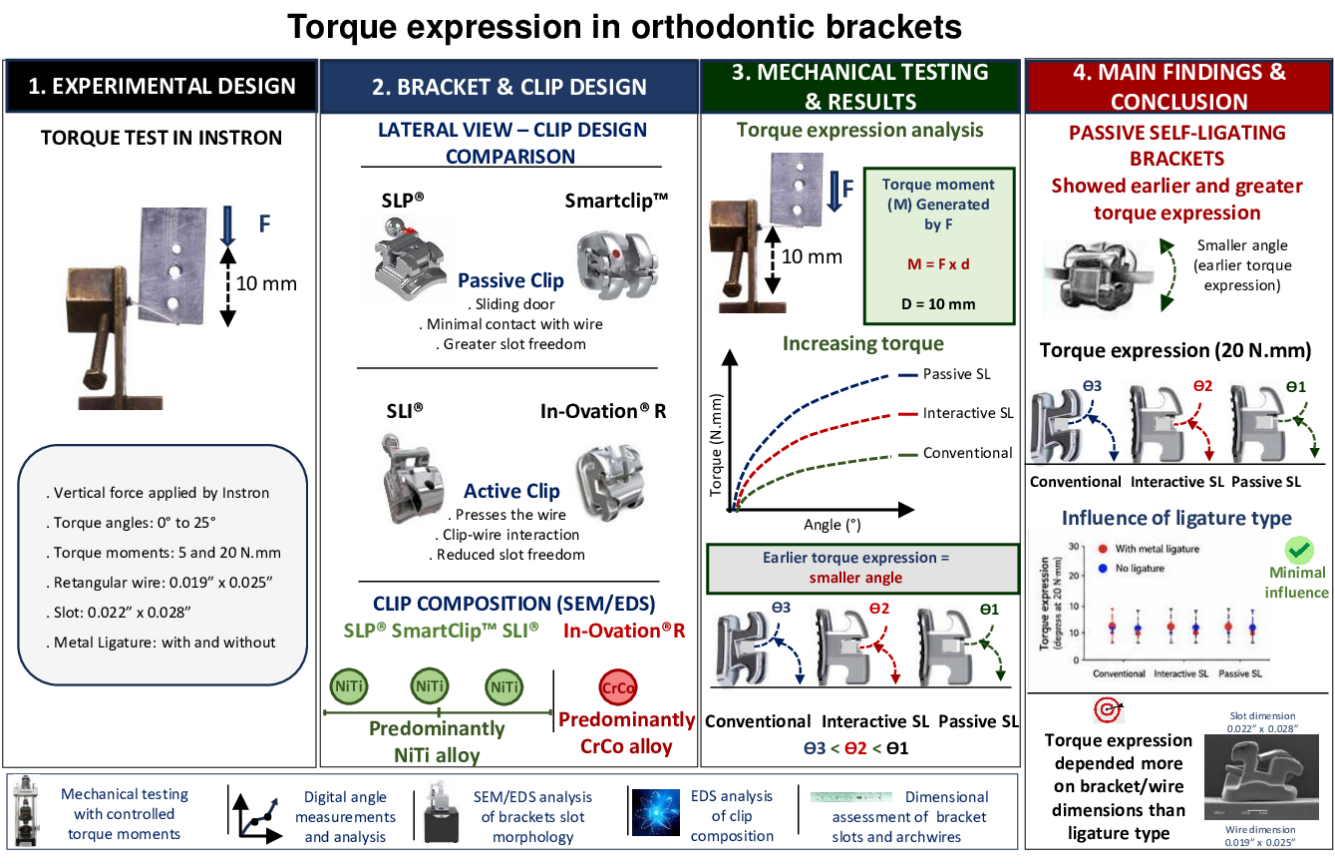

## Introduction

Torque is defined as the twist of a rectangular wire that generates a moment of force when inserted into the bracket slot ^1^ ^,^ ^2^ ^,^ ^3^ ^,^ ^4^ ^,^ ^5^. In orthodontics, torque represents the buccolingual inclination of a tooth, and achieving proper inclination at the end of treatment is essential for an appropriate occlusal relationship, an aesthetic smile line, and long-term stability ^1^ ^,^ ^2^ ^,^ ^3^ ^,^ ^4^ ^,^ ^5^. Inadequate torque control, particularly in the maxillary incisors, has been associated with compromised esthetics and may contribute to relapse of incisal inclination after orthodontic treatment.

Previous studies have also shown significant discrepancies between theoretical and clinical torque expression in bracket-archwire systems designed to control buccolingual inclination (1-6). These discrepancies are largely attributed to mechanical factors, including wire-slot play, dimensional tolerances of brackets and archwires, and variations in ligation systems. Consequently, insufficient torque expression may lead to suboptimal tooth positioning and negatively affect treatment outcomes.

During orthodontic treatment, changes in crown angulation due to torque are influenced by many factors, including archwire stiffness and size, bracket design, the gap between archwires and slots, positioning of the brackets in relation to the shape of the outer surface of the tooth, bracket deformation, and the ligation system ^1^ ^,^ ^2^ ^,^ ^3^ ^,^ ^5^ ^,^ ^7^ ^,^ ^8^ ^,^ ^9^ ^,^ ^10^ ^,^ ^11^ ^,^ ^12^ ^,^ ^13^. In this context, the composition and surface morphology of the brackets, along with slot measurements, play an essential role as they directly affect the mechanical properties, bond strength, and overall performance. Surface morphology analysis by scanning electron microscopy (SEM) reveals key features, including texture, roughness, and wear patterns, which are important for assessing the bracket's performance under mechanical stress. Energy dispersive X-ray spectroscopy (EDS) provides elemental composition data, offering insight into corrosion resistance and material stability. Additionally, precise slot measurements are essential for assessing torque application, slot parallelism, and dimension accuracy, which directly influence orthodontic treatment efficiency ^1^ ^,^ ^2^ ^,^ ^3^ ^,^ ^5^ ^,^ ^7^ ^,^ ^8^ ^,^ ^9^ ^,^ ^10^ ^,^ ^11^ ^,^ ^12^ ^,^ ^13^.

Archwires are typically attached to brackets using elastomeric, metal, or clip ligatures. Clips are used in self-ligating brackets, whereas elastomeric ligatures are commonly used in conventional bracket systems. However, these ligatures may increase friction and prevent the archwire from fully seating in the base of the bracket slot, thereby reducing torque expression efficiency. In such situations, metal ligatures may be preferred because they provide greater force to press the archwire toward the base of the slot, thereby improving torque delivery ^2^ ^,^ ^5^. Previous studies have shown that conventional brackets exhibit greater torque expression than self-ligating ones ^2^ ^,^ ^3^ ^,^ ^5^ ^,^ ^7^. Moreover, interactive self-ligating brackets may produce higher torque than passive types because their clips apply pressure on larger archwires, whereas passive clips essentially convert the bracket into a tube ^1^ ^,^ ^2^ ^,^ ^4^ ^,^ ^8^. 

The minimal friction between self-ligating brackets and archwires, resulting from clip-based closure systems rather than elastic or metal ligation, is advantageous during the early stages of alignment and leveling, enabling smaller, biologically acceptable forces. However, there may be limitations during the orthodontic treatment retraction and finishing stages, where torque control and slot expression are essential ^7^ ^,^ ^10^. In these situations, clinicians may opt for metal ligatures to improve the seating of the archwire into the base of the bracket slot, thereby enhancing torque expression in the anterior region. 

Despite previous studies addressing torque behavior in various bracket systems, a comprehensive analysis comparing torque expression at different angulations among conventional, interactive, and passive self-ligating brackets, with or without metal ligatures, remains lacking. A better understanding of how the ligation system between the archwire and different types of brackets affects torque can help clinicians choose the most suitable devices. Moreover, few studies have correlated mechanical findings with the bracket surface morphology and elemental composition, both of which may significantly influence torque expression and clinical performance.

Therefore, the present study aimed to ^1^ evaluate torque expression at different angulations on conventional brackets and interactive and passive self-ligating brackets, either ligated or not, with the use of additional metal ligatures, and ^2^ assess the surface morphology and semiquantitative chemical composition (%) of the brackets using SEM/EDS. The null hypothesis stated that the torque expression would not differ between bracket types and ligation systems.

## Materials and methods

**Figure 1:**
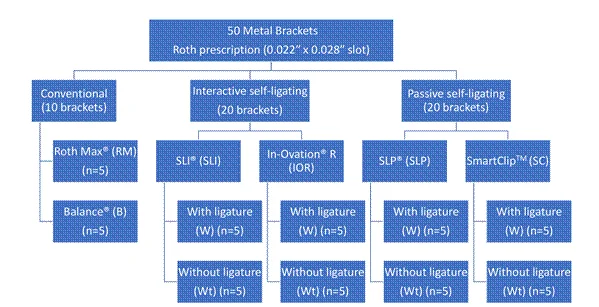
Distribution of experimental groups. All conventional brackets were exclusively tested with metal ligatures. Ten groups were tested: (G1) RMW; (G2) BW; (G3) SLIW; (G4) SLIWt; (G5) IORW; (G6) IORWt; (G7) SLPW; (G8) SLPWt; (G9) SCW; and, (G10) SCWt.

 A total of fifty metal brackets designed for right maxillary central incisors (Roth prescription and 0.022″ x 0.028″ slot) were divided into three groups according to bracket type: conventional (Roth Max^®^ [Morelli, Sorocaba, São Paulo, Brazil] and Balance^®^ [Dentsply GAC, New York, NY, USA]); interactive self-ligating (SLI^®^ [Morelli, Sorocaba, São Paulo, Brazil] and In-Ovation^®^ R [Dentsply GAC, New York, NY, USA]); and, passive self-ligating (SLP^®^ [Morelli, Sorocaba, São Paulo, Brazil] and SmartClip^TM^ [3M Unitek, St Paul, Minneapolis, Minnesota, USA]) (Figure 1). 

All conventional brackets (n=5 each) were exclusively tested with metal ligatures. The self-ligating brackets (interactive and passive) were divided into two subgroups (n=5 each) according to the ligation system, with and without metal ligatures, totaling ten groups as follows: Roth Max^®^ with ligature, Balance^®^ with ligature, SLI^®^ with ligature, SLI^®^ without ligature, In-Ovation^®^ R with ligature, In-Ovation^®^ R without ligature, SLP^®^ with ligature, SLP^®^ without ligature, SmartClip^TM^ with ligature, and SmartClip^TM^ without ligature (Figure 1). The sample size (n = 5) was defined based on previous in vitro torque expression studies with similar experimental designs and statistical power ^2^ ^,^ ^3^ ^,^ ^7^.

**Figure 2:**
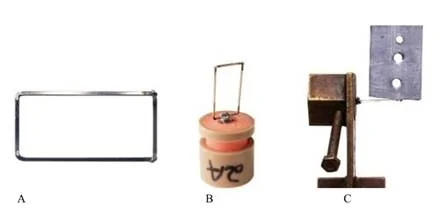
A. 0.019" x 0.025" rectangular archwire bent into a rectangular shape (20 mm x 10 mm) with two spot welds. B. Rectangular bracket-wire set ligated with a metal ligature. C. The set is fixed on the Instron universal testing machine.

Five hundred sections of rectangular stainless-steel wire with 0.019″ x 0.025″ dimensions (Morelli, Sorocaba, São Paulo, Brazil) were bent into a 20 mm x 10 mm rectangular shape and welded twice at one end (Figure 2A). The 0.019″ × 0.025″ archwire dimension was selected because it is commonly used during the finishing stage of orthodontic treatment, allowing evaluation of torque expression under clinically relevant wire-slot play conditions rather than using a full-size archwire that would minimize slot clearance. The rectangular bend design (20 mm × 10 mm) was used to standardize the lever arm length and ensure consistent torque generation during mechanical testing.

All brackets were fixed to round acrylic cylinders (22 mm high, 20 mm in diameter) with cyanoacrylate-based glue (Super Bonder Gel^®^; Loctite, Hartford, USA). The brackets were bonded on a flat surface prepared on the acrylic cylinder to ensure a stable bonding area, avoiding placement on the curved lateral surface. Brackets were positioned centrally on the acrylic cylinders, with the slot oriented parallel to the horizontal reference plane and perpendicular to the cylinders' long axes to avoid angular deviations during testing. The rectangular archwire segments were then ligated with 0.010″ metal ligatures on all conventional brackets and half of the other self-ligating brackets (Figure 2B). The other half was tested without metal ligatures. 

The cylinders with brackets and ligated archwire segments were fixed onto a universal testing machine (model 4411; Instron Corp., Canton, MA, USA). A vertical force was applied to the free end of the archwire, generating a 10-mm displacement (Figure 2C) ^(2,3)^. The crosshead speed of the testing machine was set at 1 mm/min during all measurements.

The obtained data were tabulated, and the torque angles were accurately calculated for each bracket/archwire using the following formula: 

T = Torque in N·mm 

F = Applied force in N

d = Machine displacement in mm

D = Archwire length

I = Initial height from the center to the tip of the archwire

**Figure:**
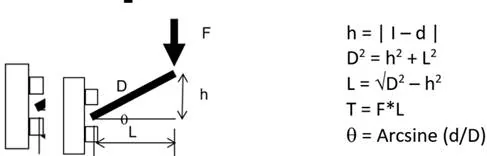

Ten sections of orthodontic archwires were tested for each bracket, totaling 50 torque readings per bracket type and manufacturer, with and without a ligature. 

Based on the previously described formula, the mean torque values (N·mm ) were recorded at specified torque angles (0°, 5°, 10°, 15°, 20°, and 25°). Additionally, by setting the torque to 5 N·mm and 20 N·mm, which correspond to the lower and upper limits of the clinically effective torque range described in the literature ^1^, the torque angles were calculated using the same formula. Smaller torque angles indicated greater torque expression of the bracket ^3^. These values were selected to identify brackets with earlier torque expression, corresponding to clinical stages in which torque control becomes critical during orthodontic finishing.

### Scanning electron microscopy (SEM/EDS)

**Figure 3:**
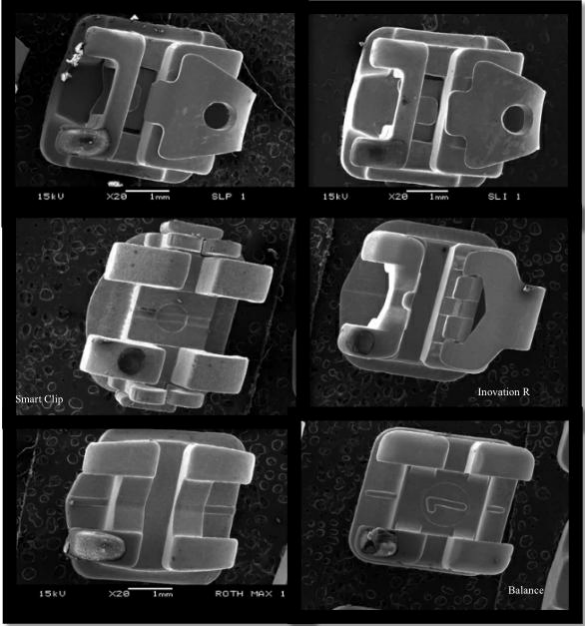
Surface morphology (frontal view) of passive (SLP^®^ and SmartClip^TM^), interactive (SLI^®^ and In-Ovation^®^ R), and conventional (Roth Max^®^ and Balance^®^) self-ligating brackets at 20x magnification (SEM).

**Figure 4:**
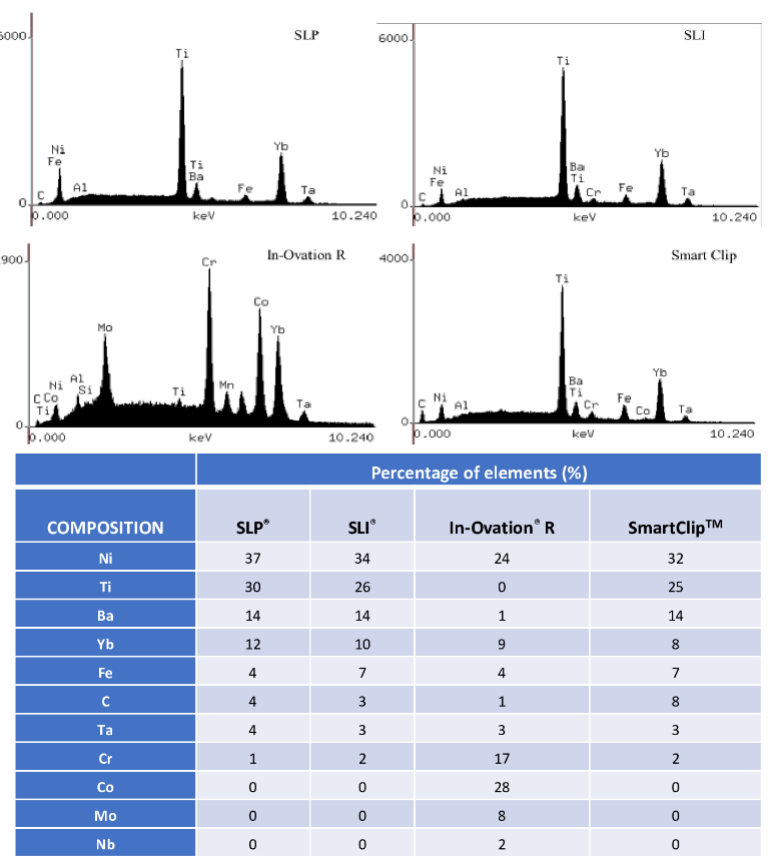
Energy-dispersive spectroscopy (SEM/EDS) and percentage of chemical elements present in passive (SLP^®^ and SmartClip^TM^) and interactive self-ligating bracket clips (In-Ovation^®^ R and SLI^®^).

Three brackets (n=3) were examined under a scanning electron microscope (EVO 40, Carl Zeiss, Cambridge, UK) at 20x magnification to assess their surface morphology (frontal view) (Figure 3). The percentage of chemical elements on the passive and interactive bracket clips was determined using energy dispersive spectroscopy (EDS), which is an SEM feature that investigates chemical composition (Figure 4). 

**Figure 5:**
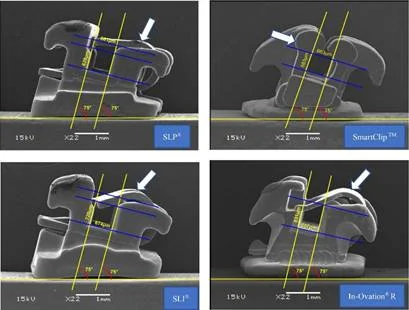
Surface morphology (lateral view) of passive and interactive self-ligating brackets at 22x magnification. The white arrow indicates the bracket clip type.

To accurately assess the angle formation and slot measurements of the brackets’ lateral surface, lines and dots were marked on the microscopes at 22x magnification using the Image J software (National Institutes of Health, Bethesda, MD, USA), which indicated the torque applied and the accuracy of slot parallelism and slot height of orthodontic accessories parallel to the SEM beam. These measurements were taken by the same operator and were modified according to earlier studies ^9^ ^,^ ^14^ (Figure 5).

### Statistical analysis

After verifying data normality and homogeneity, the torque data (N·mm) were subjected to a two-way analysis of variance (ANOVA). One-way ANOVA was applied to the angle data corresponding to torque values of 5 N·mm and 20 N·mm. Tukey's test was used to compare groups in all analyses, with a significance level of 5% (α = 0.05). All statistical analyses were performed using SPSS software (version 21; IBM Corp., Armonk, NY, USA). Slot measurements and image analyses were performed using ImageJ software (version 1.53; National Institutes of Health, Bethesda, MD, USA).

## Results

### Torque (N·mm)

The mean torque values (N·mm) required to achieve specified angles (0°, 5°, 10°, 15°, 20°, and 25°) using different metal brackets, with or without metal ligatures, are presented in Table 1. No statistically significant differences in torque among bracket types with or without ligation were observed at 0° and 5° (p > 0.05). 

At 10°, the torque of the SmartClip^TM^ without ligature (16.19 ± 4.60) was statistically higher than that of the other groups (p < 0.05), except for SmartClip^TM^ with ligature (12.08 ± 5.09). SmartClip^TM^ with ligature also showed significantly higher torque than the other groups (p < 0.05), except for In-Ovation^®^ R with ligature (5.73 ± 0.42). 

**Table 1:** Mean torque ± standard deviation (N·mm) at specified angles of 0° to 25° with 0.019” x 0.025” archwire for different metal bracket systems, with or without ligature.

Groups	Torque (N·mm)					
	0^o^	5°	10°	15°	20°	25°
G1- Roth Max^®^ with ligature (RMW)	0.50 ± 0.02 aC	1.76 ± 0.32 aC	3.49 ± 1.08 cC	6.05 ± 2.14 cC	17.58 ± 3.73 cB	31.14 ± 3.54 cA
G2- Balance^®^ with ligature (BW)	0.57 ± 0.06 aD	3.44 ± 0.16 aCD	4.97 ± 0.32 cCD	7.61 ± 0.51 cC	17.41 ± 0.75 cB	26.38 ± 2.40 cA
G3- In-Ovation^®^ R with ligature (IORW)	0.48 ± 0.38 aD	4.25 ± 0.50 aCD	5.73 ± 0.42 bcCD	9.48 ± 1.50 cC	18.55 ± 2.00 cB	26.43 ± 3.99 cA
G4- In-Ovation^®^ R without ligature (IORWt)	0.16 ± 0.04 aD	0.48 ± 0.11 aD	0.59 ± 0.13 cD	7.08 ± 1.20 cC	17.90 ± 0.93 cB	29.17 ± 0.91 cA
G5- SLI^®^ with ligature (SLIW)	0.59 ± 0.12 aB	1.64 ± 0.41 aB	2.42 ± 0.54 cB	3.76 ± 0.83 cB	6.05 ± 1.64 dB	12.67 ± 2.20 dA
G6- SLI^®^ without ligature (SLIWt)	0.15 ± 0.11 aD	0.61 ± 0.26 aD	3.06 ± 1.92 cD	9.61 ± 4.73 cC	18.45 ± 6.38 cB	28.78 ± 7.07 cA
G7- SLP^®^ with ligature (SLPW)	0.28 ± 0.05 aA	2.84 ± 0.14 aA	3.78 ± 0.18 cA	4.28 ± 0.25 cA	4.68 ± 0.32 dA	5.76 ± 0.87 eA
G8- SLP^®^ without ligature (SLPWt)	0.03 ± 0.01 aC	0.13 ± 0.19 aBC	1.19 ± 1.16 cBC	3.47 ± 1.87 cABC	5.78 ± 1.93 dAB	8.34 ± 2.36 deA
G9- SmartClip^TM^ with ligature (SCW)	0.74 ± 0.20 aE	6.37 ± 1.84 aDE	12.08 ± 5.09 abD	19.73 ± 8.74 bC	30.07 ± 8.16 bB	41.80 ± 8.25 bA
G10- SmartClip^TM^ without ligature (SCWt)	0.88 ± 0.83 aE	5.71 ± 3.88 aE	16.19 ± 4.60 aD	28.74 ± 4.95 aC	41.46 ± 5.53 aB	51.58 ± 4.50 aA

Conventional brackets - Roth Max^®^ (Morelli) and Balance^®^ (Dentsply GAC); Interactive self-ligating brackets - In-Ovation^®^ R (Dentsply GAC) and SLI^®^ (Morelli); Passive self-ligating brackets - SLP^®^ (Morelli) (Morelli) and SmartClip^TM^ (3M Unitek). Significant differences were detected for bracket type (p<0.000) and angle (p<0.000). The interaction between factors was significant (p < 0.001). Different lowercase letters in the column and uppercase letters in the row indicate statistically significant differences (α = 0.05).

From 15 to 25°, SmartClip^TM^ without a ligature had statistically higher torque than the other brackets (p < 0.05), ranging from 28.74 to 51.58 N·mm. Overall, SmartClip^TM^ brackets, particularly without ligature, demonstrated greater torque values across most evaluated angles.

The comparison among the six angulations demonstrated that conventional brackets, interactive brackets with or without ligature, and SmartClip^TM^ with and without ligature showed statistically higher torque at 25° than at 0°, 5°, 10°, 15°, and 20° (p < 0.05). 

No statistical difference was found for SLI^®^ with ligature among 0°, 5°, 10°, 15°, and 20° (p > 0.05). Similarly, SLP^®^ with ligature showed no statistically significant difference in torque at any evaluated angle (p > 0.05). 

SLP^®^ without a ligature showed statistically higher torque at 25° than at 0°, 5°, and 10° (p < 0.05). 

### Torque expression (5 N·mm and 20 N·mm ) 

The mean torque angles (°) required to achieve torque expression equivalent to 5 N·mm and 20 N·mm using different brackets are shown in Table 2. In summary, at a torque expression of 5 N·mm, SLP^®^ with ligature showed the highest mean torque angles (22.60° ± 2.30) compared with conventional brackets, SLI^®^ without ligature, In-Ovation^®^ R, and SmartClip^TM^ (with and without ligatures) (p < 0.05). The lowest torque angle was observed for SmartClip^TM^ without ligature (4.60° ± 2.10) (p < 0.05). 

Regarding the torque expression equivalent to 20 N·mm, SmartClip^TM^ with and without ligature showed the lowest angulations, which were statistically lower than those of the other groups (p < 0.001). No statistically significant differences were observed among conventional brackets, In-Ovation^®^ R (with or without ligature), and SLI^®^ without ligature (p > 0.05). SLI^®^ with ligature and SLP^®^ (with or without ligature) required mean angles greater than 27° to achieve torque expression of 20 N·mm, exceeding the maximum angle recorded in the present study. 

**Table 2:** Mean torque angle (°) ± standard deviation required to achieve torque expression of 5 N·mm and 20 N·mm with 0.019” x 0.025” arrchwire for the different bracket groups.

GROUPS	Torque Angle (°)	
	5 N·mm	20 N·mm
G1- Roth Max^®^ with ligature (RMW)	13.4 ± 2.6 bc	20.8 ± 2.9 a
G2- Balance^®^ with ligature (BW)	10.2 ± 0.8 c	21. 2 ± 0.4 a
G3- In-Ovation^®^ R with ligature (IORW)	7.0 ± 1.2 cd	21.2 ± 1.6 a
G4- In-Ovation^®^ R without ligature (IORWt)	14.2 ± 0.4 bc	21. 2 ± 0.4 a
G5- SLI^®^ with ligature (SLIW)	18.2 ± 2.3 ab	*
G6- SLI^®^ without ligature (SLIWt)	12.4 ± 2.9 c	20.8 ± 2.9 a
G7- SLP^®^ with ligature (SLPW)	22.6 ± 2.3 a	*
G8- SLP^®^ without ligature (SLPWt)	18.4 ± 4.3 ab	*
G9- SmartClip^TM^ with ligature (SCW)	4.0 ± 2.9 de	15.2 (4.4) b
G10- SmartClip^TM^ without ligature (SCWt)	4.6 ± 2.1 d	11.6 (1.7) b
ukey’s test	p < 0.001	p < 0.001

Conventional brackets - Roth Max^®^ (Morelli) and Balance^®^ (Dentsply GAC); Interactive self-ligating brackets - In-Ovation^®^ R (Dentsply GAC) and SLI^®^ (Morelli); Passive self-ligating brackets - SLP^®^ (Morelli) (Morelli) and SmartClip^TM^ (3M Unitek).

* Requires mean angles greater than 27° (largest angle calculated with Instron formula and records) for torque expression equivalent to 20 N·mm. Values with different lower-case letters in the same column differ statistically (p < 0.05).

### SEM/EDS

Figure 4 shows that the SLP^®^ clip was composed of 37% of Ni and 30% Ti, the SLI^®^ of 34% Ni and 26% Ti, the SmartClip^TM^ of 32% Ni and 26% Ti, whereas the In-Ovation^®^ R was composed of 17% Cr and 28% Co. Therefore, self-ligating bracket clips were predominantly composed of NiTi, except for In-Ovation^®^ R, which was mainly composed of CrCo (Figure 4). 

### SEM (slot parallelism and height)

Figure 5 shows that the inner walls of the bracket slots exhibit high alignment and parallelism. Furthermore, the slot dimensions varied among the evaluated self-ligating bracket systems.

## Discussion

Self-ligating brackets have been widely used in recent decades. However, controversies still persist regarding torque expression between self-ligating and conventional systems, as well as between interactive and passive self-ligating brackets ^1^ ^,^ ^5^ ^,^ ^8^ ^,^ ^15^ ^,^ ^16^ ^,^ ^17^. Conventional brackets demonstrated greater torque expression than self-ligating brackets, as described in previous studies ^2^ ^,^ ^7^ ^,^ ^18^, indicating that the clips did not press the archwire toward the base of the slot as metal ligatures do. However, in the case of interactive self-ligating brackets, previous studies have noted that they exhibit greater torque than passive brackets ^1^ ^,^ ^19^. Nevertheless, there is no clinical evidence of the superiority of one over the other ^8^. To date, no *in vitro* study has verified whether self-ligating brackets fixed with a metal ligature exhibit greater, equal, or lesser torque expression when compared with conventional brackets by the formula described earlier.

In this study, the choice for metal ligatures was based on previous studies that showed better torque control in conventional brackets when the archwire was secured with metal ligatures because they force the archwire towards the base of the slot and are not subject to degradation in the oral environment as occurs with elastomeric ligatures ^2^ ^,^ ^14^ ^,^ ^20^. This would not occur with self-ligating brackets because of their clip-closure system.

The literature shows clinically efficient torques range from 5 to 20 N·mm ^1^ ^,^ ^5^. The faster the brackets reach these torques, the smaller the torque angulation, the better the torque expression ^3^. 

From a clinical perspective, the torque levels evaluated in this study (5 and 20 N·mm) are commonly associated with different stages of orthodontic treatment. Lower torque levels may occur during the alignment and leveling phases, when initial control of incisor inclination begins. Higher torque values are generally required during the space closure and finishing stages, when precise buccolingual control of the incisors becomes essential to achieve proper occlusal and esthetic outcomes. Therefore, evaluating how different bracket-archwire-ligation combinations reach these torque levels helps better understand their potential influence on clinical torque control.

In the present study, passive self-ligating brackets, particularly SmartClipTM, reached 5 N·mm torque with smaller angulations compared with most of the other bracket systems evaluated (Table 2). This observation indicates earlier torque engagement between the archwire and bracket slot, which may contribute to more efficient torque expression. Therefore, the null hypothesis was rejected.

Unlike the findings of the present study, Brauchli et al. (2012) ^8^ found no statistically significant differences among conventional, interactive self-ligating, and passive self-ligating brackets in torque expression up to 22.6 N·mm. The literature, therefore, remains controversial, as other investigations have also reported different sequences of torque expression depending on bracket design and experimental conditions^3^.

Regarding torque expression at higher levels, passive self-ligating brackets also demonstrated earlier achievement of 20 N·mm torque compared with most other systems evaluated. However, some bracket systems did not reach this torque level within the maximum evaluated angulation (27°).

From a clinical standpoint, bracket systems that do not reach higher torque values within the tested angulation range may require additional biomechanical compensation. In such situations, orthodontists may need to incorporate torque bends in the archwire, select brackets with different torque prescriptions, or use archwires with larger dimensions or different alloys to achieve adequate buccolingual control of incisors.

Some of the differences noted above could be explained by the different methodological tests used to measure torque expression, composition, bracket type and design, size, and archwire composition and types across the several studies. 

Using a 0.019″ × 0.025″ archwire, the present study showed that different bracket systems required different torque angulations to achieve the same torque values. This finding reinforces the importance of considering the interaction between bracket slot dimensions and archwire geometry when interpreting torque expression results. Previous investigations have also reported wide variations in the torque angles required to achieve specific torque levels depending on bracket-archwire combinations ^7^ ^,^ ^21^.

Many studies ^2^ ^,^ ^7^ ^,^ ^9^ ^,^ ^10^ ^,^ ^11^ ^,^ ^14^ ^,^ ^13^ ^,^ ^15^ ^,^ ^20^ ^,^ ^21^ ^,^ ^22^ ^,^ ^23^ ^,^ ^24^ ^,^ ^25^ ^,^ ^26^ that used various bracket types and manufacturers reported oversized slots that did not comply with the manufacturers’ instructions. Also, the upper and lower walls of the slots were divergent ^9^ ^,^ ^10^ ^,^ ^11^ ^,^ ^25^ ^,^ ^26^. Some studies also showed that rectangular archwires had rounded rather than straight edges ^6^ ^,^ ^7^ ^)^ and could be undersized or oversized ^14^. Combining these factors further increases the gap between archwires and brackets, significantly reducing clinical torque expression.

In the present study, no statistically significant difference was observed between self-ligating brackets tested with and without metal ligatures. This finding may be explained by the fact that the 0.019” × 0.025” archwire already occupies a large portion of the 0.022″ slot, allowing the clip mechanism of self-ligating brackets to maintain sufficient contact between the archwire and the slot walls. Consequently, the additional force generated by metal ligatures may not significantly reduce the remaining wire-slot play or increase torque transmission under these experimental conditions. These results suggest that the dimensional characteristics of brackets and archwires may exert a greater influence on torque expression than the type of ligation system.

Another fact that may influence torque expression is the composition of the clips in self-ligating brackets. Most *in vitro* tests are performed at room temperature. Brackets composed predominantly of nickel and titanium (NiTi) may behave differently under intraoral conditions because NiTi alloys are temperature-sensitive. Conversely, clips made mainly of chromium-cobalt (CrCo) alloys may be less affected by temperature variations. In this study, most self-ligating bracket clips were composed predominantly of NiTi. In contrast, the In-Ovation^®^ R bracket was mainly composed of CrCo, as indicated by energy-dispersive spectroscopy (SEM/EDS) (Figure 4). Therefore, differences between laboratory conditions and the oral environment may influence the torque expression observed clinically.

No statistically significant difference was observed between self-ligating and conventional brackets concerning torque expression, confirming previous research findings ^2^ ^,^ ^3^ ^,^ ^4^ ^,^ ^8^. However, in the present study, SmartClip^TM^ passive bracket (with and without ligature) and In-Ovation^®^ R interactive bracket with ligature showed higher torque expression at 5 N·mm than conventional and other self-ligating brackets. A similar trend was observed at 20 N·mm for the SmartClip^TM^ passive bracket. The inner walls of the self-ligating bracket slots demonstrate no parallelism variations, as seen in Figure 4. This finding contrasts with other studies ^9^ ^,^ ^10^ ^,^ ^11^, which have reported significant variations in parallelism among most, if not all, bracket types. Furthermore, the dimensions of the slots in various self-ligating bracket systems (Figure 4) showed considerable discrepancies relative to the manufacturers' specifications, consistent with previous studies ^9^ ^,^ ^10^ ^,^ ^11^.

A previous study ^24^ evaluated the mechanical response of orthodontic bracket-archwire systems during simulated torque and observed that the mechanical characteristics of the system influence torque transmission and the effectiveness of tooth movement. Clinically, a rigid bracket should be chosen for prolonged torque ^15^. Nonetheless, several factors influence torque during orthodontic treatment, including the torque magnitude, alloy type, and thickness of rectangular archwires, archwire geometry, engineering tolerances of archwires and brackets, slot dimensions, manufacturing processes, bracket design, clip composition, bracket positioning, and tooth positioning. These variables collectively affect how torque is transmitted and expressed during treatment ^3^ ^,^ ^6^ ^,^ ^8^ ^,^ ^9^ ^,^ ^14^ ^,^ ^16^ ^,^ ^17^ ^,^ ^20^ ^,^ ^21^ ^,^ ^27^ ^,^ ^28^. 

To compensate for the gap between archwires and bracket slots and achieve more predictable buccolingual tooth inclination, orthodontists may apply specific torques and bends to archwires or select bracket prescriptions and ligation methods based on the diagnosis and treatment plan for each case. 

However, the present study has some limitations. The experimental design was conducted under in vitro conditions that do not fully replicate the complexity of the oral environment, including temperature variations, saliva, masticatory forces, and cyclic mechanical loading. Additionally, the present study evaluated only one archwire dimension (0.019″ × 0.025″) and one tooth type (maxillary central incisor), which may limit extrapolation of the findings to other clinical situations. Variations among manufacturers in bracket design and slot dimensions may also influence torque expression. Furthermore, some bracket systems (G5-SLI® with ligature, G7-SLP® with ligature, and G8-SLP® without ligature) did not achieve torque expression equivalent to 20 N·mm across the tested angulation range, preventing the calculation of the corresponding torque angle (Table 2). This limitation may be associated with the mechanical characteristics of these bracket systems and the experimental setup adopted in the present study.

Future studies should be conducted under the simulated oral environment with variable sizes and compositions of rectangular archwires. Furthermore, a larger number of bracket systems and models should be evaluated, given their influence on torque expression, as observed in both previous and the present studies. It is important to note that although there is a numerical difference between the groups (see Tables 1 and 2), the statistical tests did not identify this difference as significant, suggesting that further investigation may be required.

These findings suggest that bracket systems capable of reaching clinically relevant torque levels with smaller angulations may facilitate earlier torque engagement during orthodontic treatment. Clinicians should also be aware that dimensional discrepancies between bracket slots and rectangular archwires may significantly influence torque transmission. Therefore, careful consideration of bracket design, slot dimensions, and archwire characteristics is essential when planning orthodontic mechanics. However, these findings should be interpreted with caution, as in vitro results may not fully replicate the biomechanical conditions in the oral environment.

Passive self-ligating brackets, either ligated or not, showed the earliest torque expression at 5 and 20 N·mm under the experimental conditions evaluated. The dimensional characteristics of bracket slots and rectangular archwires appear to have a greater influence on torque expression than the type of ligation system employed. The clips of the self-ligating brackets are predominantly composed of nickel and titanium, except for the interactive self-ligating bracket, which is mainly composed of chromium and cobalt.

## Data Availability

The research data are available upon request
